# Prospects and Potential Uses of Genomic Prediction of Key Performance Traits in Tetraploid Potato

**DOI:** 10.3389/fpls.2018.00159

**Published:** 2018-03-07

**Authors:** Benjamin Stich, Delphine Van Inghelandt

**Affiliations:** ^1^Institute for Quantitative Genetics and Genomics of Plants, Heinrich Heine University, Düsseldorf, Germany; ^2^Cluster of Excellence on Plant Sciences, From Complex Traits towards Synthetic Modules, Düsseldorf, Germany

**Keywords:** genomic prediction, tetraploid potato, *Phytophthora infestans*, maturity, tuber starch content, tuber yield

## Abstract

Genomic prediction is a routine tool in breeding programs of most major animal and plant species. However, its usefulness for potato breeding has not yet been evaluated in detail. The objectives of this study were to (i) examine the prospects of genomic prediction of key performance traits in a diversity panel of tetraploid potato modeling additive, dominance, and epistatic effects, (ii) investigate the effects of size and make up of training set, number of test environments and molecular markers on prediction accuracy, and (iii) assess the effect of including markers from candidate genes on the prediction accuracy. With genomic best linear unbiased prediction (GBLUP), BayesA, BayesCπ, and Bayesian LASSO, four different prediction methods were used for genomic prediction of relative area under disease progress curve after a *Phytophthora infestans* infection, plant maturity, maturity corrected resistance, tuber starch content, tuber starch yield (TSY), and tuber yield (TY) of 184 tetraploid potato clones or subsets thereof genotyped with the SolCAP 8.3k SNP array. The cross-validated prediction accuracies with GBLUP and the three Bayesian approaches for the six evaluated traits ranged from about 0.5 to about 0.8. For traits with a high expected genetic complexity, such as TSY and TY, we observed an 8% higher prediction accuracy using a model with additive and dominance effects compared with a model with additive effects only. Our results suggest that for oligogenic traits in general and when diagnostic markers are available in particular, the use of Bayesian methods for genomic prediction is highly recommended and that the diagnostic markers should be modeled as fixed effects. The evaluation of the relative performance of genomic prediction vs. phenotypic selection indicated that the former is superior, assuming cycle lengths and selection intensities that are possible to realize in commercial potato breeding programs.

## Introduction

Despite the important role of potato for securing world-wide human nutrition (FAOSTAT, 2015), potato breeding realized in the last 50 years annual gains from selection that were considerably lower than those realized for other crop species (Douches et al., 1996). The tetraploidy and heterozygosity of potato are considered to be reasons for that (Jansky et al., 2016). Furthermore, the high number of selection criteria (Barocka and Ross, 1985) which can be evaluated in many cases only in the later stages of the breeding program on the harvest product, the tuber, is considered to be another factor limiting gain from selection. Marker-assisted selection approaches, however, have the potential to increase the gain from selection (Gebhardt, 2013). Although many quantitative trait loci (QTL) have been identified (for review see Gebhardt et al., 2014), the impact of marker-assisted selection for improving truly quantitative traits in potato breeding is limited (Slater et al., 2016). This is attributed to the low proportion of variance explained by most of the identified QTL as well as the fact that many identified QTL are specific to a particular phenotyping environment or genetic background.

Genomic prediction provides an alternative method to use genomic information in breeding decisions (Meuwissen et al., 2001). Instead of using only significant marker-trait associations to build up the prediction model, genomic prediction uses all markers simultaneously (Windhausen et al., 2012). This approach has been evaluated in most major animal and plant species (for review see Desta and Ortiz, 2014) and is or is becoming a routine tool in commercial and public breeding programs. For potato, Slater et al. (2016) reported prediction accuracies between 0.19 for yield and 0.78 for maturity. In a set of interconnected biparental populations, Sverrisdóttir et al. (2017) obtained cross-validated prediction correlations of 0.56 and 0.73 for tuber starch content and chipping quality. Nevertheless, further research is needed to investigate whether such high prediction accuracies are also observed for other traits as well as in genetic material that was not derived from systematic crosses of well-chosen parental clones but corresponds to a diversity panel. Furthermore, the effect of relationship between the test set and the validation set in potato has also not been examined previously.

Most developments of genomic prediction methods were initiated in dairy cattle with the aim of selecting sires with high breeding value (cf. Meuwissen et al., 2001). Thus, prediction models were developed to account for the contribution of additive effects to phenotypic traits, whereas nonadditive effects were typically not considered (de Almeida Filho et al., 2016). However, in clonally propagated plant species that typically show a high degree of heterozygosity, considering dominance effects in the genomic prediction model has the potential to improve predictions. Furthermore, epistasis potentially contributes to the genetic variation of quantitative traits (Mackay, 2014). With few exceptions in tree breeding (Muñoz et al., 2014; de Almeida Filho et al., 2016), genomic selection approaches considering additive, dominance, and epistatic effects have not been evaluated in a field crop context.

The objectives of this study were to (i) examine the prospects of genomic prediction of key performance traits in a diversity panel of tetraploid potato modeling additive, dominance, and epistatic effects, (ii) investigate the effects of size and make up of training set, number of test environments, and molecular markers on prediction accuracy, and (iii) assess the effect of including markers from candidate genes on the prediction accuracy.

## Materials and methods

### Plant material, phenotypic evaluation, and genomic data

Our study was based on 184 tetraploid potato clones, subsequently designated as PIN184 population, described previously by Pajerowska-Mukhtar et al. (2009). In brief, the population consisted of 96 clones, which were important genitors but mainly advanced breeding clones from each of the breeding programs of Boehm-Nordkartoffel-Agrarproduktion OHG (Ebstorf, Germany) (BNA subset) and SaKa Pflanzenzucht GmbH & Co. KG (Windeby, Germany) (SaKa subset). The clones represented all market usages, but late clones with a plant maturity score < 4 were excluded. Eight clones were included in both subsets. As described in detail by Pajerowska-Mukhtar et al. (2009), both subsets were evaluated in 3 years each at one location for the area under disease progress curve (AUDPC) after *Phytophthora infestans* infection and for plant maturity (PM). PM was scored from 1 to 9, where 1 indicates very late and 9 very early maturity. For each year^*^location combination, which was in the following designated as environment, trait values for rAUDPC were calculated as the ratio of each clone's AUDPC and the environmental mean. As AUDPC and rAUDPC were highly correlated, we report only results obtained for the latter. Furthermore, from AUDPC and PM, the trait maturity corrected resistance (MCR) was calculated (Pajerowska-Mukhtar et al., 2009). At each of the six environments, tuber starch content (TSC) was assessed. For the BNA subset, information on tuber yield (TY) has been collected in three environments which allowed together with the TSC the calculation of tuber starch yield (TSY=TSC^*^TY). This assessment was performed in an experiment, which was laid out as a randomized block design, with one replication per environment, where each plot had two rows with eight plants each. As described by Mosquera et al. (2016), the PIN184 population was genotyped using the 8.3 k SolCAP potato genotyping array (Hamilton et al., 2011). For each SNP locus, one of the five possible genotypes (AAAA, AAAB, AABB, ABBB, or BBBB) was manually assigned to the individuals using the GenomeStudio Software version 2011.1 (Illumina) as described by Stich et al. (2013). Furthermore, for a total of 85 candidate loci for *P. infestans* resistance, one to several amplicons have been Sanger sequenced for the PIN184 population (Gebhardt et al., 2004; Pajerowska-Mukhtar et al., 2009; Odeny et al., 2010; Muktar et al., 2015; Mosquera et al., 2016). The genotypic data for the 6052 SolCAP SNPs as well as the 1,010 SNPs from the 85 candidate gens was kindly provided by Christiane Gebhardt, MPI for Plant Breeding Research.

In order to examine the prediction accuracy of our models in unrelated genetic material, we included in our study 187 tetraploid clones from the potato diversity panel for which phenotypic data are kindly provided by the SolCAP consortium and which correspond to those evaluated by Rosyara et al. (2016). For these clones, which were subsequently designated as SolCAP187 population, genotypic information from the 8.3 k SolCAP array as well as phenotypic information on vine maturity 95/120 days after planting were available.

After removing all markers with a minor allele frequency <0.05, the proportion of missing values was about 1% across the two SNP data sets. Such a level warrants the imputation of missing values by using the median allele frequency for each SNP.

### Statistical analyses

#### Genetic relationship between clones

The results of Slater et al. (2014) indicated the minor effect of double reduction on heritability estimates in potato across a range of traits and, thus, was neglected in our study. The additive relationship matrix was calculated in accordance with method 1 of VanRaden (2008) from the SNP-clone matrix, where SNPs were coded in an additive autotetraploid way (Slater et al., 2016). The dominance relationship matrix was calculated according to Nishio and Satoh (2014). Additive-additive, additive-dominance, and dominance-dominance epistasis relationship matrices were calculated from the Hadamard product of the respective additive and dominance relationship matrices (VanRaden, 2008).

Associations among the clones were revealed with principal component (PC) analyses based on allele frequency matrices considering the allele dosage information. The number of columns of these matrices correspond to the number of marker loci and the number of rows to the number of clones. Clones were grouped by k-means clustering to *n* = 4 and 8, where random individuals were chosen as the initial values of the centroids. This procedure was repeated 100 times for each number of clusters, and the clustering that was observed with the highest frequency was used for our analyses (Supplementary Material). The molecular variance among and within clusters (Gst) was assessed (Gerlach et al., 2010). Bi-locus linkage disequilibrium (LD) was characterized by the LD measure *r*^2^ (Hill and Robertson, 1968), which was calculated for all pairs of SNPs from the SolCAP array that mapped to the same chromosome.

#### Variance components and heritability

Since eight clones were in common between the BNA and SaKa subsets of the PIN184 population, we performed a joint data analysis for both subsets using the following statistical model, where each year^*^location combination was treated as an environment:

(1) $$\begin{array}{r} y_{ij}=\mu +g_{i}+l_{j}+e_{ij}, \end{array}$$

where *y*_*ij*_ was the entry mean for the *i*th clone in the *j*th environment, μ was an intercept term, *g*_*i*_ was the effect of the *i*th clone, *l*_*j*_ was the effect of the *j*th environment, and *e*_*ij*_ was the residual. As the environments comprised two purposefully selected locations, the environmental effects *l*_*j*_ were regarded as fixed. On the basis of best linear unbiased estimation, adjusted entry means for each clone were derived in each set of environments (e = 6, 5, 4, 3, or 2), applying model [1] considering the clone effects as fixed. For estimation of variance components, *g*_*i*_ was considered as random. Heritability on an entry mean basis was calculated as $h^{2}=\sigma_{g}^{2}/\left(\sigma_{g}^{2}+\bar{w}/2\right),$ where $\sigma_{g}^{2}$ was the genetic variance and $\bar{w}$ the mean variance of the difference between two adjusted entry means (Holland, 2003).

The genetic variance among and within clusters (Qst) (Prout and Barker, 1993; Spitze, 1993) was estimated by partitioning the clone effect in model [1] into the effect of the cluster and that of the clone nested within the cluster.

#### Genomic prediction models

Despite the fact that PM was scored on an ordinal scale, the QQplots of the adjusted entry means across all environments as well as per environment did not indicate a deviation from the normal distribution as well as the residuals were normally distributed. Therefore, the adjusted entry mean of each clone of the PIN184 population for PM was predicted using the same four prediction methods as for rAUDPC, MCR, and TSC as well as the clones of the BNA subset for TSC, TY, and TSY: Genomic best linear unbiased prediction (GBLUP), BayesA, BayesCπ, and Bayesian LASSO (BL). Details of these methods were described previously (Meuwissen et al., 2001; Park and Casella, 2008; Hayes et al., 2009; Habier et al., 2011) and will not be listed here. GBLUP method was used as implemented in the R package sommer (Covarrubias-Pazaran, 2016), where the residuals assumed to be normally distributed with mean 0 and variance $\sigma_{r}^{2}$. BayesA, BayesCπ, and BL were fitted using the R package BGLR (de los Campos and Perez-Rodriguez, 2016) with default hyperparameter values described previously (de los Campos et al., 2013; Pérez and de los Campos, 2014). In all, 30,000 Markov chain Monte Carlo iterations were used, of which the first 10,000 were discarded as burn-in and every third sample was kept for parameter estimation.

For each of the above mentioned statistical models, different genetic models were examined: Model M1 considered only additive effects, M2 additive and dominance effects, whereas model M3 considered additive, dominance, and the three types of epistatic effects (e.g., Stich and Gebhardt, 2011). We observed in a previous study that polymorphisms in the *StAOS2* locus explained individually between 30 and 40% of the phenotypic variance (Pajerowska-Mukhtar et al., 2009). Therefore, we examined variants of models M1, M2, and M3 where the diagnostic SNP691 from the *StAOS2* locus was considered as fixed or random covariable. These variants of model M^*^, were designated as M^*^CF or M^*^CR, respectively. Different sets of SNPs were used for the above described genomic predictions: (i) all 6052 SolCAP SNPs, (ii) in 100 independent runs each, the SolCAP SNPs were stratified sampled in such a way that a genome-wide equal distribution of SNPs was obtained with a density of 5, 1.33, 0.67, and 0.13 Mbp^−1^, (iii) all SNPs from the 85 candidate genes for *P. infestans* resistance, and (iv) all SolCAP SNPs and the 85 candidate genes for *P. infestans* resistance.

The genomic prediction of genotypic values is of interest in our study. Therefore, we calculated the prediction accuracy [*r*(ĝ, *g*)] as the Pearson correlation between the phenotype and the genomic estimated genotypic values divided by the square root of heritability *h*^2^ of the target trait evaluated in the respective set of environments.

#### Genomic prediction cross-validation schemes

The standard scheme for validation of genomic prediction was five-fold cross-validation. For this purpose, the clones of population PIN184 or the BNA subset were randomly subdivided into five disjoint subsets. One subset was left out for validation, whereas the other four subsets were used as training set. This procedure was replicated 20 times, yielding a total of 100 cross-validation runs.

Different analyses were performed to evaluate the effect of different factors (F) on genomic prediction: (F1) the sample size of the training set was varied; (F2) the number of test environments in which the training and validation set were evaluated was varied; (F3) the effect of “reduced” relationship between training and validation set was examined: (F3A) performance of one half of the clones in one cluster was predicted based on all the clones of the remaining clusters. This procedure was replicated 20 times. In each replication, a different set of genotypes was placed into the two halves of the cluster. (F3B) performance of one half of the clones in one cluster was predicted from a combination of the clones from the remaining clusters, and the other half of the clones in the considered cluster, where the number of clones in the remaining clusters, was reduced by sampling such that the total number of clones in the training set was the same as in F3A. This procedure was repeated 20 times as described for F3A. Due to the high computational effort of the Bayesian methods, these analyses as well as the simulations of different sets of SNPs, which were described above, were performed only with the prediction method GBLUP.

If not stated differently, all statistical analyses were performed using statistical software R version 3.3.2 (R Development Core Team, 2016).

## Results

### Heritability

For the PIN184 population, which consists of 184 tetraploid elite potato clones, phenotypic information on four quantitative traits has been assessed across six location^*^year combinations. We observed for the heritability on an entry mean basis *h*^2^ high values of 0.7 for rAUDPC and MCR and very high values >0.9 for PM and TSC (Table 1). For the BNA subset, which was evaluated in three environments also for TY in addition to the above mentioned characters, high heritabilities close to 0.8 were observed for TSY and TY.

**Table 1:** Means of relative area under disease progress curve (rAUDPC), plant maturity (PM), maturity corrected resistance (MCR), tuber starch content (TSC), tuber starch yield (TSY), and tuber yield (TY), their genetic variance ($\sigma_{g}^{2}$), and broad-sense heritability *h*^2^ estimated for 184 tetraploid potato clones (PIN184) or a subset of 96 clones thereof (BNA subset).

	**PIN184**				**BNA subset**		
	**rAUDPC**	**MCR**	**PM**	**TSC**	**TSC**	**TSY**	**TY**
	**[rel.]**	**[rel.]**	**[rating 1–9]**	**[%]**	**[%]**	**[dt/ha]**	**[dt/ha]**
Mean	0.37	3.9^*^10^−3^	5.5	16.8	17.7	105	599
$\sigma_{g}^{2}$	7.1^*^10^−3^	4.5^*^10^−3^	1.52	7.2	8.3	296	9323
*h*^2^	0.77	0.68	0.92	0.92	0.95	0.78	0.77

### Genetic relationship and LD

**Figure 1:**
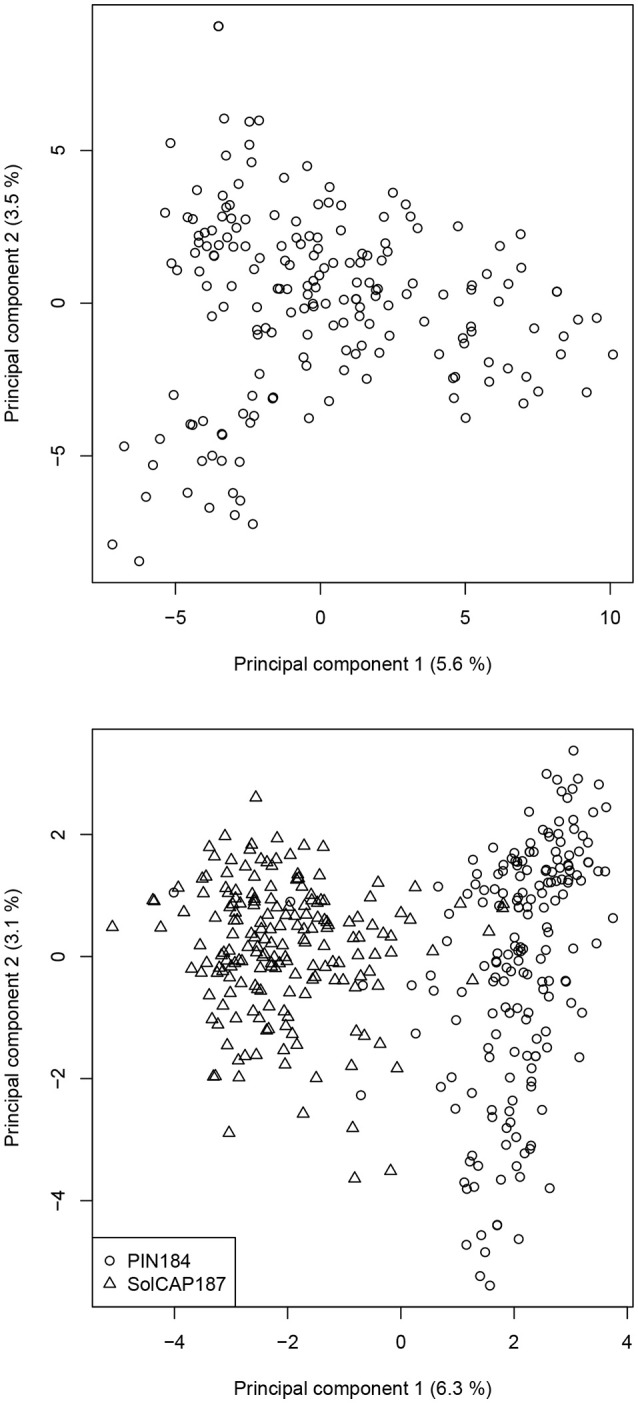
Principal component (PC) analysis based on the polymorphic SolCAP SNPs for the PIN184 population **(upper)** and the PIN184 and SolCAP187 populations together **(lower)**. Numbers in parentheses refer to the proportion of variance explained by the PC.

**Figure 2:**
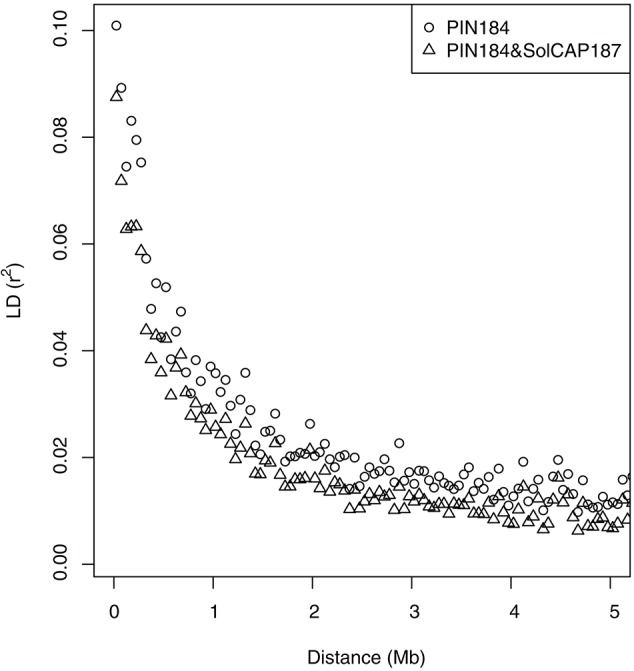
Decay of linkage disequilibrium (LD, r^2^) with distance between pairs of SNPs. LD averaged over chromosomes is given in distance bins of 50 kb.

In the PC analysis based on allele frequency estimates of all clones of the PIN184 population, the first two PCs explained 5.6 and 3.5% of the molecular variance (Figure 1). PC1 and PC2 showed a tendency to separate in two to three clusters which were in some accordance with the market usage of the corresponding potato clones (data not shown). The PC analysis of the PIN184 and the SolCAP187 population together revealed two distinct clusters. The first cluster was mainly made up of clones from the PIN184 population, whereas the second cluster comprised mainly clones from the SolCAP187 population. LD and LD decay per chromosome were estimated using the physical map positions of the SolCAP SNPs. The proportion of pairwise *r*^2^ values >0.1 and >0.8 across all SNP pairs mapping to the same chromosome was 1.5 and 0.01 %, respectively, in the PIN184 population. The mean *r*^2^ value of pairs of SNPs with a distance < 10kb was about 0.1 (Figure 2). The mean *r*^2^ value between adjacent markers was 0.11. For the combined set of clones from PIN184 and SolCAP187, the extent of LD was lower than that for PIN184 alone.

### A comparison of prediction methods and genetic models

When using all SolCAP SNPs for genomic prediction, in the PIN184 population cross validated prediction accuracies around 0.8 were observed for TSC and MCR (Table 2). The prediction accuracies for rAUDPC and PM were with 0.65 lower. For the BNA subset, which comprised about half of the clones of PIN184, prediction accuracies around 0.5 were observed for TSY and TY. In contrast to the above described differences among traits, only marginal differences were observed between the different prediction methods, when relying on the SolCAP SNPs.

**Table 2:** Median and standard deviation of prediction accuracy [*r*(ĝ, *g*)] of genomic prediction in PIN184 and the BNA subset obtained with the prediction methods GBLUP, BayesA, and Cπ, as well as Bayesian LASSO (BL) under the genetic models M1, M2, and M3, for different SNP sets across 100 cross-validation runs.

**Prediction**	**Genetic**	**PIN184 (*****n*** = **184)**				**BNA subset (*****n*** = **96)**		
**method**	**model**	**rAUDPC**	**MCR**	**PM**	**TSC**	**TSC**	**TSY**	**TY**
**ALL SolCAP SNPs**								
GBLUP	M1	0.66 ± 0.11	0.76 ± 0.12	0.65 ± 0.09	0.82 ± 0.06	0.86 ± 0.07	0.46 ± 0.19	0.50 ± 0.20
	M2	0.69 ± 0.11	0.76 ± 0.14	0.66 ± 0.09	0.81 ± 0.07	0.86 ± 0.15	0.49 ± 0.19	0.54 ± 0.17
	M3	0.68 ± 0.18	0.75 ± 0.16	0.66 ± 0.12	0.72 ± 0.37	0.39 ± 0.40	0.45 ± 0.18	0.51 ± 0.20
BayesA	M1	0.69 ± 0.11	0.76 ± 0.12	0.71 ± 0.09	0.82 ± 0.05	0.87 ± 0.06	0.50 ± 0.18	0.53 ± 0.21
	M2	0.68 ± 0.10	0.75 ± 0.12	0.68 ± 0.08	0.83 ± 0.05	0.84 ± 0.08	0.46 ± 0.18	0.52 ± 0.20
BayesCπ	M1	0.69 ± 0.11	0.75 ± 0.11	0.66 ± 0.08	0.83 ± 0.04	0.86 ± 0.08	0.45 ± 0.20	0.54 ± 0.20
	M2	0.66 ± 0.13	0.76 ± 0.13	0.63 ± 0.10	0.81 ± 0.06	0.84 ± 0.08	0.45 ± 0.19	0.49 ± 0.20
BL	M1	0.67 ± 0.11	0.75 ± 0.11	0.65 ± 0.09	0.83 ± 0.06	0.85 ± 0.07	0.50 ± 0.21	0.54 ± 0.20
	M2	0.69 ± 0.12	0.77 ± 0.12	0.63 ± 0.08	0.82 ± 0.05	0.86 ± 0.07	0.40 ± 0.25	0.47 ± 0.22
**ALL SolCAP SNPs AND** ***StAOS2*** **SNP691 AS RANDOM EFFECT**								
GBLUP	M1CR	0.68 ± 0.13	0.83 ± 0.13					
	M2CR	0.67 ± 0.12	0.83 ± 0.13					
BayesA	M1CR	0.86 ± 0.06	0.86 ± 0.10					
	M2CR	0.86 ± 0.08	0.88 ± 0.12					
BayesCπ	M1CR	0.70 ± 0.11	0.75 ± 0.11					
	M2CR	0.71 ± 0.10	0.78 ± 0.12					
BL	M1CR	0.72 ± 0.12	0.77 ± 0.12					
	M2CR	0.71 ± 0.12	0.78 ± 0.12					
**ALL SOLCAP SNPs and** ***StAOS2*** **SNP691 AS FIXED EFFECT**								
GBLUP	M1CF	0.67 ± 0.11	0.81 ± 0.12					
	M2CF	0.66 ± 0.11	0.82 ± 0.13					
BayesA	M1CF	0.85 ± 0.08	0.88 ± 0.07					
	M2CF	0.84 ± 0.08	0.88 ± 0.11					
BayesCπ	M1CF	0.85 ± 0.06	0.89 ± 0.10					
	M2CF	0.83 ± 0.08	0.89 ± 0.09					
BL	M1CF	0.85 ± 0.07	0.88 ± 0.11					
	M2CF	0.85 ± 0.08	0.87 ± 0.11					
**ALL SNPs FROM 85 CANDIDATE LOCI FOR** ***P. infestans*** **RESISTANCE**								
GBLUP	M2	0.70 ± 0.09	0.78 ± 0.12	0.66 ± 0.11	0.71 ± 0.08	0.70 ± 0.11	0.31 ± 0.20	0.55 ± 0.20
BayesA	M2	0.85 ± 0.08	0.87 ± 0.12	0.67 ± 0.09	0.81 ± 0.06	0.66 ± 0.14	0.32 ± 0.23	0.47 ± 0.21
BayesCπ	M2	0.71 ± 0.11	0.79 ± 0.13	0.64 ± 0.08	0.81 ± 0.05	0.66 ± 0.12	0.29 ± 0.21	0.43 ± 0.21
BL	M2	0.71 ± 0.10	0.79 ± 0.10	0.67 ± 0.08	0.83 ± 0.06	0.67 ± 0.14	0.33 ± 0.20	0.48 ± 0.21
**ALL SOLCAP SNPs AND THE 85 CANDIDATE LOCI FOR** ***P. infestans*** **RESISTANCE**								
GBLUP	M2	0.73 ± 0.10	0.78 ± 0.14	0.68 ± 0.08	0.80 ± 0.06	0.86 ± 0.13	0.44 ± 0.19	0.52 ± 0.19
BayesA	M2	0.79 ± 0.08	0.82 ± 0.09	0.63 ± 0.09	0.72 ± 0.07	0.83 ± 0.08	0.42 ± 0.22	0.50 ± 0.21
BayesCπ	M2	0.78 ± 0.10	0.85 ± 0.09	0.64 ± 0.09	0.72 ± 0.07	0.83 ± 0.08	0.44 ± 0.21	0.52 ± 0.20
BL	M2	0.78 ± 0.09	0.84 ± 0.11	0.63 ± 0.09	0.63 ± 0.09	0.84 ± 0.07	0.48 ± 0.19	0.55 ± 0.18

*The considered traits were relative area under disease progress curve (rAUDPC), plant maturity (PM), maturity corrected resistance (MCR), tuber starch content (TSC), tuber starch yield (TSY), and tuber yield (TY)*.

For the GBLUP prediction method, three genetic models with additive (M1), additive and dominance (M2), as well as a model with additive, dominance, and epistatic effects (M3) were examined. Across the six traits, we observed the lowest prediction accuracy for the genetic model with additive, dominance, and epistatic effects (M3, Table 2). The prediction accuracies observed for the model with additive effects (M1) was only slightly lower compared to the model considering additive and dominance effects (M2). However, especially for TSY and TY, the difference was more pronounced than for TSC. This trend, observed for the GBLUP prediction method, was not observed for the three Bayesian prediction methods examined in our study. For BayesA, Cπ, and BL, the M2 model showed for >70 % of the examined scenarios a lower prediction accuracy compared with the M1 model.

### Genomic prediction for traits with available diagnostic SNPs

We observed in a previous study that polymorphisms in the *StAOS2* locus explained individually between 30 and 40% of the phenotyic variance of rAUDPC and MCR. Therefore, we examined the prediction of these traits not only by using the SolCAP SNPs, but also by adding SNP 691 of the *StAOS2* locus as a covariable. Adding that SNP as a fixed or random effect improved the prediction accuracy across all prediction methods and traits (Table 2). For BayesCπ and BL, the consideration of the diagnostic SNP as a fixed effect resulted in higher prediction accuracies compared with the modeling as a random effect. For GBLUP and BayesA, the prediction accuracies of models with a random effect for the diagnostic SNP were slightly higher than those with a fixed effect. The use of all SolCAP SNPs together with the SNPs from the 85 candidate loci resulted in no improvement of the prediction accuracy, compared to the scenario when all SNP from the SolCAP array and the diagnostic SNP were used (Table 2). A scenario in which the prediction was based only on SNPs from the 85 candidate loci led only to a slight reduction of the prediction accuracy for rAUDPC, MCR, and PM, but a considerable reduction for TSC, TSY, and TY compared with the scenario when all SolCAP SNPs and the diagnostic SNP were used.

### Effects of sample size, number of test environments and SNPs, as well as relatedness on prediction accuracy

For the GBLUP prediction method, we observed a 10% reduction of the prediction accuracy for the traits rAUDPC, MCR, and PM, when reducing the size of the training set from 147 to 74, whereas for TSC this reduction was only 5% (Table 3). The same trend was observed for the genetic models M1 and M3 (data not shown). For the scenario with a fixed effect for the diagnostic SNP, we observed no reduction of the prediction accuracy for rAUDPC but a 10% reduction for MCR. The reduction of the number of test environments from 6 to 2 did not result in a reduction of the prediction accuracy for any of the examined traits.

**Table 3:** Median and standard deviation of prediction accuracy [*r*(ĝ, *g*)] of genomic prediction in PIN184 obtained with the prediction method GBLUP under the genetic models M2 (all SolCAP SNPs) and M2CF (all SolCAP SNPs and SNP691 from *StAOS2* locus), with different numbers of clones *(n)* and environments *(e)* in which the training and/or validation set were evaluated across 100 cross-validation runs.

**Genetic**	**Training set**		**Validation set**		**PIN184**			
**model**	***n***	***e***	***n***	***e***	**rAUDPC**	**MCR**	**PM**	**TSC**
**VARIATION OF THE SAMPLE SIZE OF THE TRAINING SET—F1**									
M2	147	6	37	6	0.69 ± 0.11	0.76 ± 0.14	0.66 ± 0.09	0.81 ± 0.07
	110	6	74	6	0.65 ± 0.08	0.71 ± 0.08	0.63 ± 0.07	0.78 ± 0.07
	74	6	110	6	0.59 ± 0.07	0.64 ± 0.08	0.59 ± 0.04	0.77 ± 0.08
M2CF	147	6	37	6	0.66 ± 0.11	0.82 ± 0.13		
	110	6	74	6	0.67 ± 0.06	0.78 ± 0.09		
	74	6	110	6	0.69 ± 0.09	0.74 ± 0.09		
**VARIATION OF THE NUMBER OF TEST ENVIRONMENTS—F2**									
M2	147	6	37	6	0.69 ± 0.11	0.76 ± 0.14	0.66 ± 0.09	0.81 ± 0.07
	147	5	37	5	0.71 ± 0.12	0.71 ± 0.13	0.66 ± 0.09	0.81 ± 0.06
	147	4	37	4	0.67 ± 0.14	0.65 ± 0.16	0.62 ± 0.10	0.81 ± 0.08
	147	3	37	3	0.62 ± 0.15	0.74 ± 0.22	0.67 ± 0.10	0.81 ± 0.09
	147	2	37	2	0.73 ± 0.17	0.73 ± 0.18	0.76 ± 0.13	0.78 ± 0.12

*The considered traits were relative area under disease progress curve (rAUDPC), plant maturity (PM), maturity corrected resistance (MCR), and tuber starch content (TSC)*.

**Figure 3:**
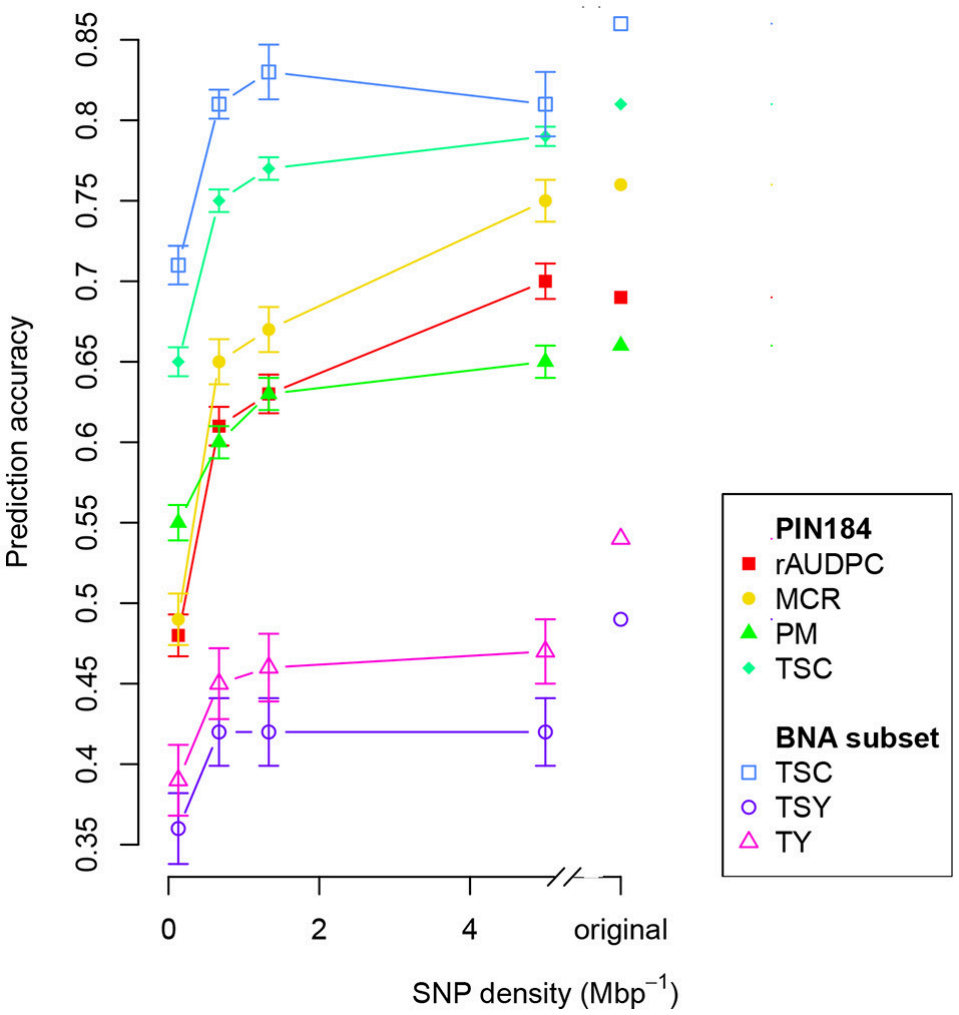
Prediction accuracy within PIN184 and the BNA subset obtained with GBLUP using additive and dominance relationship matrices (model M2) calculated from different numbers of SNPs. Shown are the median values for all traits obtained from 100 cross-validation runs. The considered traits were relative area under disease progress curve (rAUDPC), plant maturity (PM), maturity corrected resistance (MCR), tuber starch content (TSC), tuber starch yield (TSY), and tuber yield (TY). Vertical bars depict the standard error.

The number of SolCAP SNPs corresponds to a genome-wide SNP density of 8.3 Mbp^−1^ that is unequally distributed across the genome. When decreasing this density to a genome-wide equally distributed density of 5, a reduction of the prediction accuracy of about 5% was observed (Figure 3). A linear trend of a reduced prediction accuracy was observed down to a SNP density of about 1 Mbp^−1^. For even lower SNP densities, the decrease of the prediction accuracy became exponential.

The prediction accuracy for performance in a specific cluster using marker effects estimated in the other clusters, ranged between 0.31 for MCR and 0.66 for TSC with high standard deviations (Table 4, F3A). No consistent trend across the examined traits was observed when increasing the number of clusters from 4 to 8. When 50% of the genotypes in the validation set were included in the training set, the prediction accuracy increased for all traits by about the same extent (Table 4, F3B).

**Table 4:** Median and standard deviation of prediction accuracy [*r*(ĝ, *g*)] of genomic prediction in PIN184 obtained with the prediction method GBLUP under the genetic model M2, for all SolCAP SNPs, with a reduced relationship between training and validation set across 100 cross-validation runs.

**Clusters**	**Training set**	**Validation set**	**PIN184**			
	***n***	***n***	**rAUDPC**	**MCR**	**PM**	**TSC**
**PREDICTION OF 50% OF THE GENOTYPES IN ONE CLUSTER BASED ON ALL OTHER CLUSTERS—F3A**						
4	116–154	15–34	0.35 ± 0.17	0.31 ± 0.23	0.34 ± 0.16	0.66 ± 0.13
8	147–177	3–18	0.32 ± 0.35	0.35 ± 0.46	0.47 ± 0.32	0.51 ± 0.33
**PREDICTION OF 50% OF THE GENOTYPES IN ONE CLUSTER BASED ON ALL OTHER CLUSTERS**						
**PLUS THE OTHER 50% FROM THE SAME CLUSTER—F3B**						
4	116–154	15–34	0.41 ± 0.19^***^	0.40 ± 0.30^***^	0.37 ± 0.16^***^	0.73 ± 0.17^*^
8	147–177	3–18	0.50 ± 0.47^**^	0.48 ± 0.55^*ns*^	0.47 ± 0.25^*ns*^	0.57 ± 0.30^*ns*^

The studied traits were relative area under disease progress curve (rAUDPC), plant maturity (PM), maturity corrected resistance (MCR), and tuber starch content (TSC). Prediction accuracies for scenario F3B marked by ^ns^ or ^*^, ^**^, ^***^ are non significantly or significantly (P < 0.05, 0.01, 0.001) higher than those of the corresponding comparison in scenario F3A.

Analysis of genetic variance revealed that dividing the clones in an increasing number of clusters increased for all traits except PM the variance among populations while minimizing the variance within populations (Table 5, Qst). For the molecular variance, the same trend as for the genetic variance was observed. However, the proportion of the variance among clusters compared with the total variance was with values around 15% about one third to one fourth of that observed for the phenotypic traits (Table 5, Gst).

**Table 5:** Genetic and molecular variance among ($\sigma_{p}^{2}$) and within ($\sigma_{g\left(p\right)}^{2}$) clusters in the PIN184 population and their standard deviations.

	**4 Clusters**	**8 Clusters**
**Qst: Genetic variance**		
**rAUDPC**		
$\sigma_{p}^{2}$	0.0033 ± 0.057	0.0029 ± 0.054
$\sigma_{g\left(p\right)}^{2}$	0.0049 ± 0.070	0.0050 ± 0.071
$\sigma_{p}^{2}$/($\sigma_{g\left(p\right)}^{2}+\sigma_{p}^{2}$)	0.403	0.368
**MCR**		
$\sigma_{p}^{2}$	0.0027 ± 0.052	0.0029 ± 0.054
$\sigma_{g\left(p\right)}^{2}$	0.0030 ± 0.055	0.0028 ± 0.053
$\sigma_{p}^{2}$/($\sigma_{g\left(p\right)}^{2}+\sigma_{p}^{2}$)	0.472	0.512
**PM**		
$\sigma_{p}^{2}$	0.63 ± 0.79	0.42 ± 0.66
$\sigma_{g\left(p\right)}^{2}$	1.09 ± 1.05	1.06 ± 1.03
$\sigma_{p}^{2}$/($\sigma_{g\left(p\right)}^{2}+\sigma_{p}^{2}$)	0.376	0.288
**TSC**		
$\sigma_{p}^{2}$	2.36 ± 1.54	3.77 ± 1.94
$\sigma_{g\left(p\right)}^{2}$	5.43 ± 2.33	3.44 ± 1.85
$\sigma_{p}^{2}$/($\sigma_{g\left(p\right)}^{2}+\sigma_{p}^{2}$)	0.303	0.523
**Gst: Molecular variance**		
$\sigma_{p}^{2}$	0.094	0.124
$\sigma_{g\left(p\right)}^{2}$	0.973	0.972
$\sigma_{p}^{2}$/($\sigma_{g\left(p\right)}^{2}+\sigma_{p}^{2}$)	0.088	0.113

*The considered traits were relative area under disease progress curve (rAUDPC), plant maturity (PM), maturity corrected resistance (MCR), and tuber starch content (TSC)*.

We evaluated the accuracy of predicting vine maturity of the SolCAP187 population using the GBLUP prediction method and the PIN184 population as training set. The prediction accuracies for the different genetic models were all negative and ranged from −0.06 to −0.14.

## Discussion

### Factors influencing the prediction accuracy in genomic selection experiments

#### Prediction method

Various methods have been proposed for genomic prediction (for review see Desta and Ortiz, 2014). With GBLUP, BayesA, BayesCπ, and BL, four of these methods have been used in our study for genomic prediction in tetraploid potato. We observed only marginal differences among the prediction accuracies of different prediction methods for the same trait when using the SolCAP SNPs to estimate the additive and dominance relationship matrix (Table 2). This finding is in good agreement with earlier studies in a plant context (e.g., Lorenzana and Bernardo, 2009; Rousselle et al., 2013).

In a previous study, we identified SNPs in the gene *StAOS2* that explained between 30 and 40% of the phenotypic variance of rAUDPC and MCR (Pajerowska-Mukhtar et al., 2009). One of these SNPs was considered as a random covariable in the prediction approach. In that case, the Bayesian methods, especially BayesA, resulted in considerably higher prediction accuracies for rAUDPC and MCR than the GBLUP method. Our finding can be explained by the property of the Bayesian methods that different SNPs explain different proportions of the phenotypic variance, which is not the case for GBLUP. Furthermore, in BayesA, the assumption of a common variance across all marker effects, which is made by BayesCπ, is most strongly relaxed (Meuwissen et al., 2001). This finding suggests that for oligogenic traits in general and especially when diagnostic markers are available, the use of Bayesian methods for genomic prediction is highly recommended.

In addition, we also observed that considering the diagnostic SNP in the *StAOS2* gene as fixed effect resulted for all prediction methods except BayesA in the highest prediction accuracies (Table 2). For BayesA, the difference between considering the diagnostic SNP as fixed or random effect was marginal. These observations are supported by results of computer simulations of Bernardo (2014) and suggest that if genomic prediction is applied to traits for which diagnostic markers for major QTL are available, these markers should be considered as fixed effects, even when Bayesian models are chosen.

#### Genetic architecture of the trait

As described in detail for various crops, the prediction accuracies differed considerably among traits (e.g., Rousselle et al., 2013; Sverrisdóttir et al., 2017). However, when the heritability on an entry mean basis, calculated from the same number of plots, is used as a proxy variable for the genetic complexity of the traits under consideration (cf. Schön et al., 2004), only a weak correlation between the genetic complexity and the realized prediction ability (data not shown) or prediction accuracy for a trait was observed (Tables 1, 2). For example, the heritability for PM was among the highest observed, whereas the prediction accuracy was one of the lowest. This is consonant with the observation that for PM the relationship between expected (Daetwyler et al., 2010) and observed prediction accuracy was especially low (data not shown). One explanation for this finding is that genetic main or interaction effects (López-Fanjul et al., 2003), structural or epigenetic variants exist which cannot be well predicted with the available SNPs. The reason for this might be that the SolCAP SNPs have been discovered in elite North American potato germplasm.

Another finding that supported this explanation is that in the analysis of molecular variance, the ratio between the genetic variance among and within clusters calculated from phenotypic data (Qst) was three to four times higher than that calculated based on molecular marker data (Gst) (Table 5). This can be because the SNPs used in our study represent only a small part of the entire genotypic variability in the potato genome. In addition to SNPs, other genomic variants such as structural or epigenetic variants contribute as well to phenotypic diversity. Furthermore, these genomic variants are not necessarily in LD with the available SNPs. Strong discrepancies between Qst and Gst were also observed for other plant species (Porcher et al., 2004; Windhausen et al., 2012; Volis et al., 2015) but were the strongest for potato. This aspect warrants further research.

#### Genetic model

Potato is currently bred as a clonal species that is highly heterozygous, which allows dominance effects to contribute to phenotypic variation. In order to quantify that contribution, we examined genetic models for genome-wide prediction that considered additive and dominance effects. On average across all traits, the prediction accuracies observed for such a model were moderately higher than for the model considering additive effects alone (M1&2, Table 2). However, for traits with a high expected genetic complexity, such as TSY and TY, the difference was more pronounced than for traits with a low expected genetic complexity, such as TSC or PM. This finding illustrates the importance of including dominance effects in prediction models when selecting clones for their per-se performance as commercial products. However, as dominance effects cannot be transmitted to the next generation, only additive effects should be considered when making decisions about the utility of clones to be used as parents of new segregating populations.

Another non-additive component potentially influencing the genotypic value of genotypes is epistasis. We examined a genetic model that considered, besides additive and dominance effects, the three types of epistatic interactions. Across the six traits, we observed the lowest prediction accuracy for that model (M3, Table 2). The results of He et al. (2017) indicated a small increase in prediction accuracy when comparing a model considering epistatic interactions with one neglecting such effects in a set of winter wheat inbred lines. One potential explanation for that discrepancy is that the study of He et al. (2017) examined a diploid, autogamous species which shows a slower decay of LD compared with the rapid decay observed in this study (Figure 2). Furthermore, the population size used in our study was smaller compared with that evaluated in the study of He et al. (2017). Both factors led to a reduced estimation of epistatic variances and a lower prediction of the corresponding epistatic effects (Lorenzana and Bernardo, 2009) in our study compared with that of He et al. (2017). Therefore, in the following sections, the results of the model considering epistatic effects will not be further discussed.

#### Size and make up of the training set

The size of the training set is expected to influence the prediction accuracy (cf. Riedelsheimer et al., 2013). This trend was also observed in our study (Table 3). However, the observed rate of reduction of the prediction accuracy with reduced size of the training set was extraordinarily low. We observed a reduction of only 5% when bisecting the size of the training set. The reason for this insensitivity of the prediction accuracy to the size of the training set could be the low genetic complexity or the high extent of genotypic variance for the traits under consideration. As this is not necessarily the case for other potato germplasm, we do recommend planning future genomic prediction experiments with larger population sizes than used in our study.

Besides the size of the training set, the degree of relatedness or differentiation between training and validation set also matters. We observed a 10–15% higher prediction accuracy when predicting half of the genotypes of one cluster based on a training set that also included the other half of that cluster compared with a scenario where these related clones were not included in the training set (Table 4). However, compared with previous studies (e.g., Windhausen et al., 2012), that increase was small. This observation was unexpected, as the partitioning of genetic variance among and within clusters in the PIN184 populations revealed similar or even higher Gst and Qst values compared with that of Windhausen et al. (2012). One explanation is that despite differences in mean trait values among clusters, the loci that cause phenotypic variation in the different clusters are the same. In summary, the results indicate that even when using a germplasm set of tetraploid potato that comprises clones representing different market usages as training set for genomic prediction, the level of population differentiation is low enough to provide high prediction accuracies for validation sets of clones from other market usages.

We observed negative prediction accuracies when predicting vine maturity of the SolCAP187 population from the PM genomic prediction model of the PIN184 population. This finding can be explained by the higher differentiation between PIN184 and SolCAP187 compared with the differentiation between clusters of the PIN184 population. However, a more likely reason is that the environments in which vine maturity was assessed for the SolCAP187 population belong to a different mega-environment and, thus, reveal a very different genotype^*^environment pattern from the environments in which the PIN184 population was evaluated.

#### Number and type of molecular markers

Across all traits, we observed a linear decrease of the prediction accuracy when decreasing the SNP density from 5 to about 1 Mbp^−1^ (Figure 3). Such a linear decrease is expected only for marker densities where the relationship between the LD measure *r*^2^ and the distance between markers is approximately linear. This explanation is in agreement with the observed low extent of LD in the PIN184 population (Figure 2). These findings suggest that the prediction accuracy realized in our study is only to a small proportion due to LD between markers and QTL and mostly due to the modeling of relatedness. This explanation is supported by the observation of a 10–15% higher prediction accuracy when predicting half of the genotypes of one cluster based on a training set that also included the other half of that cluster compared with a scenario where these related clones were not included in the training set (Table 4). Furthermore, this is also supported by the finding, that the highest prediction accuracy was observed for the original SNP density of 8.4 Mbp^−1^ which is more than 10 times lower than the distance in which for 98.5% of the loci pairs, the *r*^2^ value drops below 0.1. Therefore, we expect that by increasing the marker density to a level so that the mean *r*^2^ values between adjacent markers reaches values >0.20 (Calus et al., 2008; Habier et al., 2010) the prediction accuracy for traits such as TSY and TY can be increased considerably compared with the levels observed in our study. Based on the observed decay of LD in potato in the PIN184 population, we estimate that in the order of 200–500,000 SNPs equally distributed across the genome are required to realize such levels of *r*^2^ for diverse germplasm sets. These numbers are considerably higher than those estimated by D'hoop et al. (2010). For genomic prediction within segregating populations or across connected segregating populations (e.g., Sverrisdóttir et al., 2017), a lower SNP density might be sufficient.

For the prediction of rAUDPC, MCR, and PM based on the SNPs from 85 candidate loci for *P. infestans* resistance, we observed a higher accuracy than for the prediction only based on the SolCAP SNPs (Table 3). This observation is due to the fact that the candidate genes were very well selected as functional and positional candidates for *P. infestans* resistance and its interplay of this trait with maturity. However, for the traits TSC, TSY, and TY, the opposite trend was observed. Furthermore, the combination of SNPs from candidate genes and the SolCAP SNPs resulted in the highest prediction accuracy. Both findings illustrated the importance of representing the entire genome in genomic prediction experiments.

#### Number of test environments

We observed approximately the same prediction accuracies, regardless of the number of test environments used for the evaluation of training and validation set. Our finding suggests that the decrease in prediction ability due to a reduced number of environments is compensated by a reduced heritability. This illustrates that if the target population of environments can be represented by a lower number of environments, this leads to an increase of the relative efficiency of genomic selection which is calculated as the ratio of prediction accuracy and phenotypic accuracy (the square root of the heritability).

### Potential uses of genomic prediction in tetraploid potato breeding

In the context of quantifying the potential advantage of genomic prediction versus phenotypic selection, the former can be viewed as an indirect selection method, whereas the latter is considered as a direct selection method. The relative merit of indirect selection to direct selection per unit time can be calculated as the indirect selection response (*CR*_*X*_) divided by the direct selection response (*R*_*X*_) (Falconer and Mackay, 1996). This ratio can be arranged to the inequality:

(2) $$\begin{array}{r} L_{Y}<\frac{i_{Y}r_{A}}{i_{X}h_{X}}L_{X}, \end{array}$$

where *i*_*Y*_ is the selection intensity of the indirect selection and *i*_*X*_ the selection intensity of the direct selection. *L*_*Y*_ and *L*_*X*_ are the lengths of the indirect and direct selection cycles, respectively. *r*_*A*_ is the genetic correlation between the indirect trait ind the direct trait and corresponds in the context of genomic prediction to the prediction accuracy. *h*_*X*_ is the square root of the heritability of the direct trait. Genomic prediction is superior to phenotypic selection, if this ratio is >1. Assuming the same selection intensities for genomic prediction and phenotypic selection (*i*_*X*_ = *i*_*Y*_) and considering the *h*^2^ estimates and the prediction accuracies of our study, the maximum relative cycle length of indirect selection can be calculated for which identical selection gains are realized with direct and indirect selection. These maximum relative cycle lengths of indirect selection were 103% (MCR), 98% (rAUDPC), 85% (TSC), 73% (PM), and 60% (TSY and TY). Therefore, genomic prediction for MCR is already superior to phenotypic selection even without reducing the length of one cycle, whereas for the other traits, one cycle of genomic predictions needs to have maximally the above mentioned length relative to phenotypic selection to result in the same gain of selection. These are very promising figures, compared with the results reported for other crop species (cf. Heffner et al., 2010).

As a clonally propagated crop species, potato has a low propagation coefficient. This, however, leads to restrictions in reducing the length of one breeding cycle by skipping a single or several stages of a standard breeding scheme (Table 6) using genomic prediction. Therefore, in the context of potato breeding, we expect that the increase of *i*_*Y*_ is more promising than reducing *L*_*Y*_. After the availability of sufficiently sized training sets, it will be possible to shift resources that were previously used for phenotyping toward the creation and genotyping of considerably more clones than under phenotypic selection. This in turn allows to increase *i*_*Y*_, if ultra-low cost genotyping techniques are available.

**Table 6:** Standard potato breeding scheme and dimensioning (V. Prigge, SaKa Pflanzenzucht GmbH & Co. KG, personal communication).

**Year**	**Stage/activity**	**No. of clones**	**No. of tubers per clone in trials and multiplication**
1	Cross		
2	Pot seedling	140,000	1
3	Field seedling	90,000	1
4	A clone	5,000	10
5	B clone	600	60
6	C clone	100	300
7	D clone	30	1,200
8	Official trials 1	8	6,000
9	Official trials 2	4	20,000

The costs of current genotyping techniques are high compared with the low phenotyping costs at year 2 and 3 (V. Prigge, SaKa Pflanzenzucht GmbH & Co. KG, personal communication) of a standard potato breeding scheme (Table 6). Therefore, we consider the improvement of the selection efficiency by selecting A clones (year 4) based on genomic predictions for traits that are typically used for selection at that stage as highly relevant. However, even more interesting is the selection of A clones based on genomic predictions for traits that are typically not possible to reliably evaluate at that stage due to the need of a high number of tubers, e.g., bruising, *P. infestans* resistance, yield. The concrete allocation of resources which optimize the genetic gain of a potato breeding program using genomic selection warrants further research.

In conclusion, our results indicate that the application of genomic prediction in breeding programs for tetraploid potato has a high potential for increasing the gain from selection.

## Author contributions

BS: Conceived and designed the study; BS and DV: Analyzed the data and wrote the paper. Both authors read and approved the final manuscript.

### Conflict of interest statement

The authors declare that the research was conducted in the absence of any commercial or financial relationships that could be construed as a potential conflict of interest.
